# Indolent T cell lymphoproliferative disorder with villous atrophy in small intestine diagnosed by single-balloon enteroscopy

**DOI:** 10.1007/s12328-019-00971-1

**Published:** 2019-04-09

**Authors:** Takashi Nagaishi, Daiki Yamada, Kohei Suzuki, Ryosuke Fukuyo, Eiko Saito, Masayoshi Fukuda, Taro Watabe, Naoya Tsugawa, Kengo Takeuchi, Kouhei Yamamoto, Ayako Arai, Kazuo Ohtsuka, Mamoru Watanabe

**Affiliations:** 1 Department of Gastroenterology and Hepatology, Graduate School of Medical Science, Tokyo Medical and Dental University (TMDU), 1-5-45 Yushima, Bunkyo-ku, Tokyo, 113-8519 Japan; 2 Department of Gastric Surgery, TMDU, Tokyo, Japan; 3 Division of Pathology, The Cancer Institute, Japanese Foundation for Cancer Research, Tokyo, Japan; 4 Department of Comprehensive Pathology, TMDU, Tokyo, Japan; 5 Department of Hematological Therapeutics, Graduate School of Medical Science, TMDU, Tokyo, Japan; 6 Division of Hematology and Oncology, Department of Internal Medicine, St. Marianna University School of Medicine, Kanagawa, Japan

**Keywords:** Chronic diarrhea, Single-balloon enteroscopy, Villous atrophy, Indolent T cell lymphoproliferative disorder of the gastrointestinal tract, Mogamulizumab

## Abstract

Chronic diarrhea is one of the major symptoms in gastroenterology. However, this may be caused by pathologic conditions for which the diagnosis is critical. Villous atrophy, as an endoscopic lesion, accompanied by chronic diarrhea can occasionally be observed in the patients with inflammatory diseases of the gastrointestinal (GI) tract. Herein, we present a case with persistent diarrhea accompanied by intestinal wall thickening without any other significant endoscopic features other than villous atrophy in the jejunum and the ileum, where we diagnosed as an indolent T cell lymphoproliferative disorder (T-LPD) of the GI tract, defined in the 2016–2017 revised World Health Organization classification, via single-balloon enteroscopy (SBE). Interestingly, we found the same lymphocyte infiltration from the distal third portion of the duodenum, where gastroscopy could not reach, via SBE, even though no endoscopic findings were observed such as villous atrophy. Since infiltrating cells in the intestinal tissues were CCR4^+^, mogamulizumab was administered with resulting durable symptomatic remission for more than 2 years. Patients with persistent diarrhea may have serious small intestinal disorder including not only chronic inflammatory diseases but also lymphoid neoplasmic conditions including T-LPD of GI tract.

## Introduction

Diarrhea is one of the major symptoms in the field of gastroenterology, and many cases are caused by inflammation due to infection with pathogens such as viruses and bacteria [1]. In addition, those with long standing diarrhea are often diagnosed as irritable bowel syndrome (IBS) when other specific inflammation and/or infection are ruled out. However, diarrhea may sometimes reflect the presence of significant disorder and thus a wide differential diagnosis is critical.

Villous atrophy can occasionally be observed under endoscopic studies in patients with persistent diarrhea, especially in those suffering from inflammatory diseases of the gastrointestinal (GI) tract such as celiac disease, Crohn’s disease, eosinophilic gastroenteritis and familial Mediterranean fever [2–5]. Crohn’s disease and eosinophilic gastroenteritis usually show specific histological features such as intestinal granuloma and eosinophil infiltrate, respectively. Celiac disease and familial Mediterranean fever are more prevalent in the Western hemisphere. In addition, villous atrophy in such diseases is often accompanied by other endoscopic lesions associated with inflammation such as redness, bleeding, erosion, ulceration, stenosis or polypoid lesions.

Herein, we present a case with persistent diarrhea without any other significant endoscopic findings other than villous atrophy in the small intestine where we diagnosed one form of lymphoproliferative disease using single-balloon enteroscopy (SBE).

## Case report

A 69-year-old Japanese man with a history of anal fistula presented with diarrhea, intermittent fever and wasting. He did not have any specific family history. He had moved to a Southeast Asian country 12 years earlier, and he started having persistent diarrhea 2 years later. He came back to Japan to visit an outside hospital and was diagnosed as IBS, because stool culture and colonoscopy were normal, and the parasite infection studies were negative. Four years later, his diarrhea worsened with occasional fever. These symptoms continued to worsen for the subsequent year, and his body weight decreased 6 kg over 6 months. Gastroscopy and colonoscopy were unremarkable. However, computed tomography (CT) study of the abdomen detected jejunal wall thickening. Therefore, he was admitted to our institution for further evaluations.

**Fig. 1:**
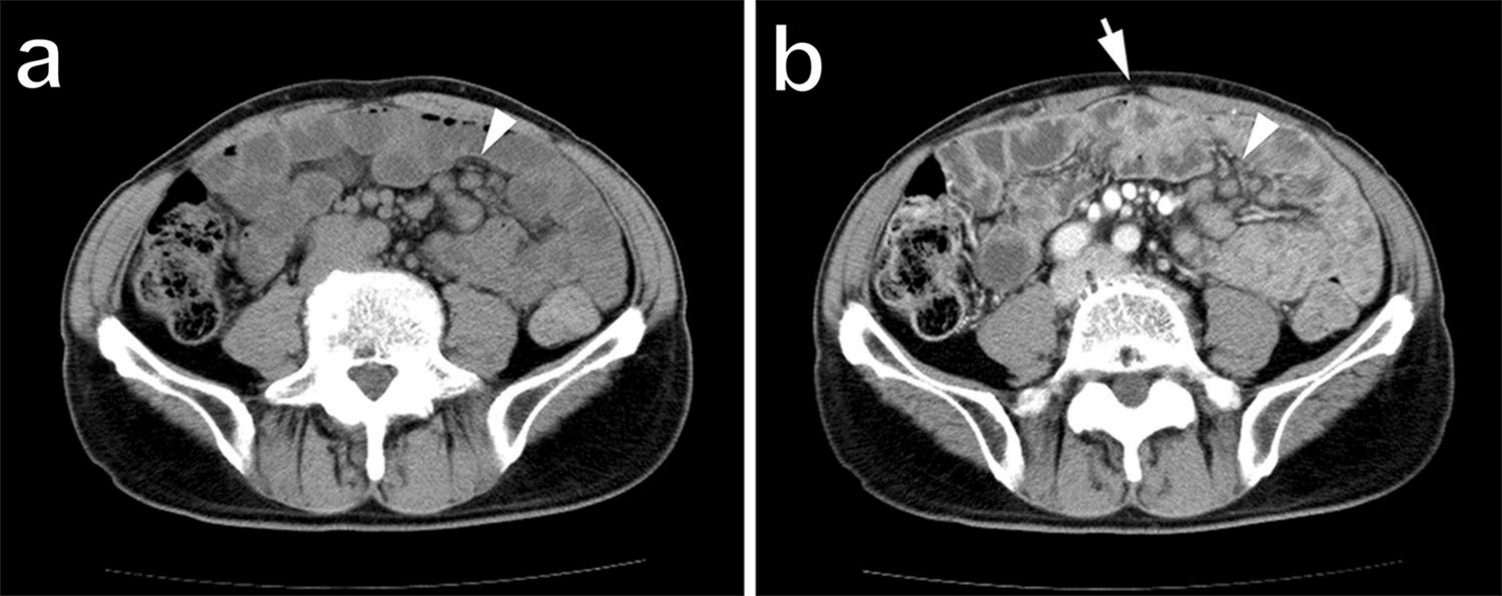
CT of pelvis without (**a**) and with contrast (**b**). Arrow and arrowhead point to the thickened wall of small intestine and the swollen lymph nodes, respectively

**Fig. 2:**
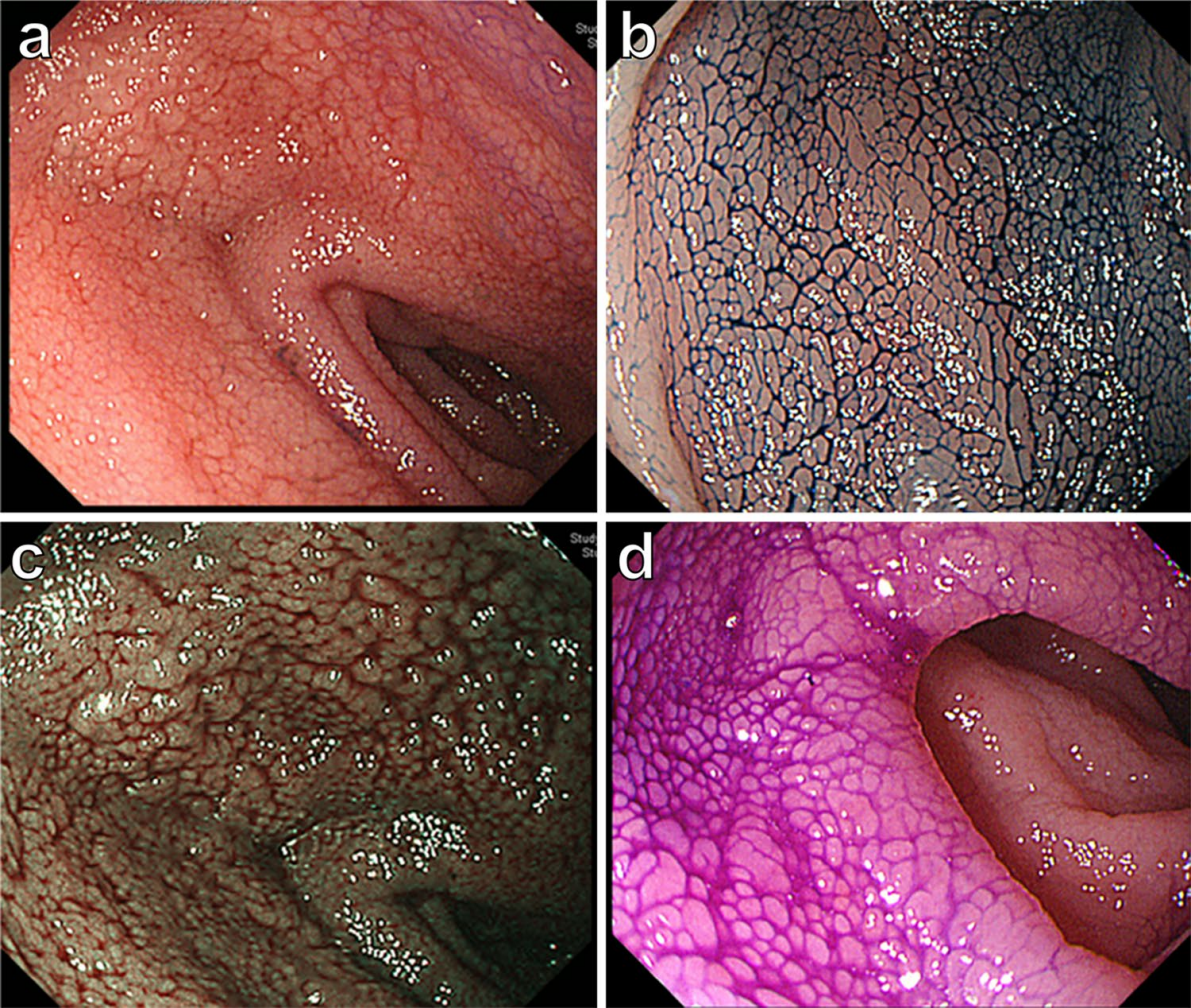
Endoscopic appearances of jejunum. Observations with normal view (**a**), indigo carmine solution (**b**), narrow band imaging (**c**), and crystal violet staining (**d**) are shown, respectively

**Fig. 3:**
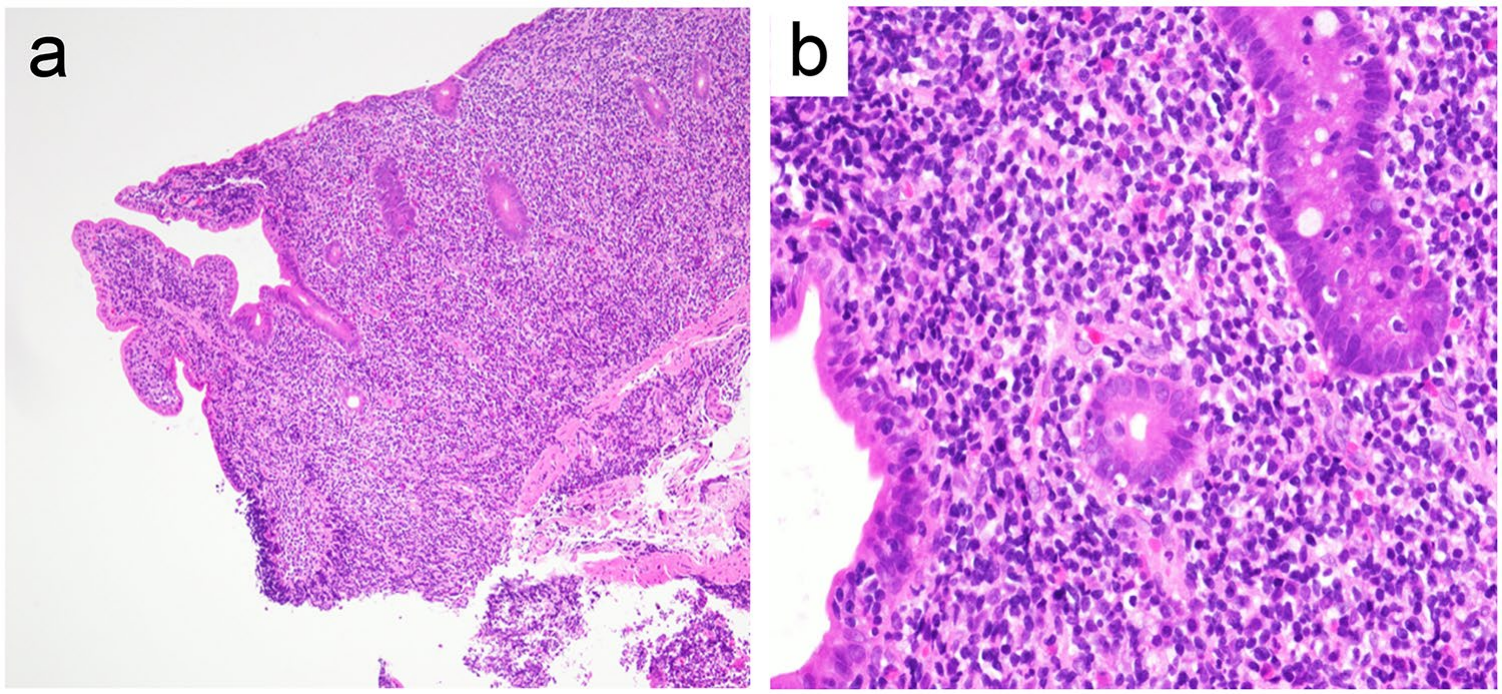
Hematoxylin and eosin (H&E)-stained histological features of SBE biopsy. Formalin-fixed, paraffin-embedded SBE biopsy specimens were sectioned, and then stained with H&E. **a** Low power view shows the loss of villous architecture and severe infiltration with small cells in the lamina propria and the submucosal layers of jejunum. **b** High power magnification reveals infiltrating mononuclear cells with mild nuclear grade

**Fig. 4:**
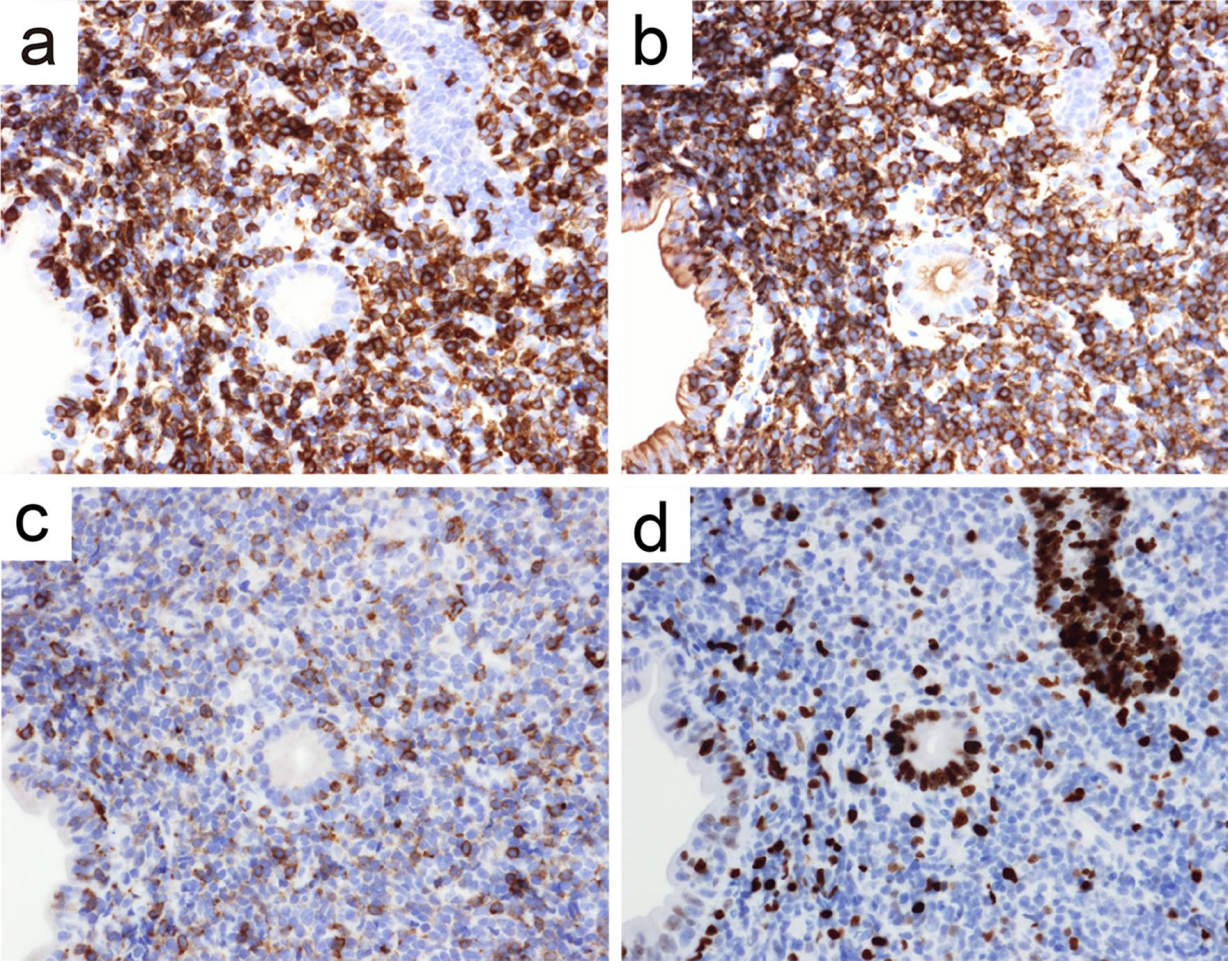
Immunophenotypical analysis of the infiltrating cells. Serial sections from Fig. 3 were stained with Abs against either CD3 (**a**), CD5 (**b**), CD7 (**c**), or Ki-67 (**d**), respectively

**Fig. 5:**
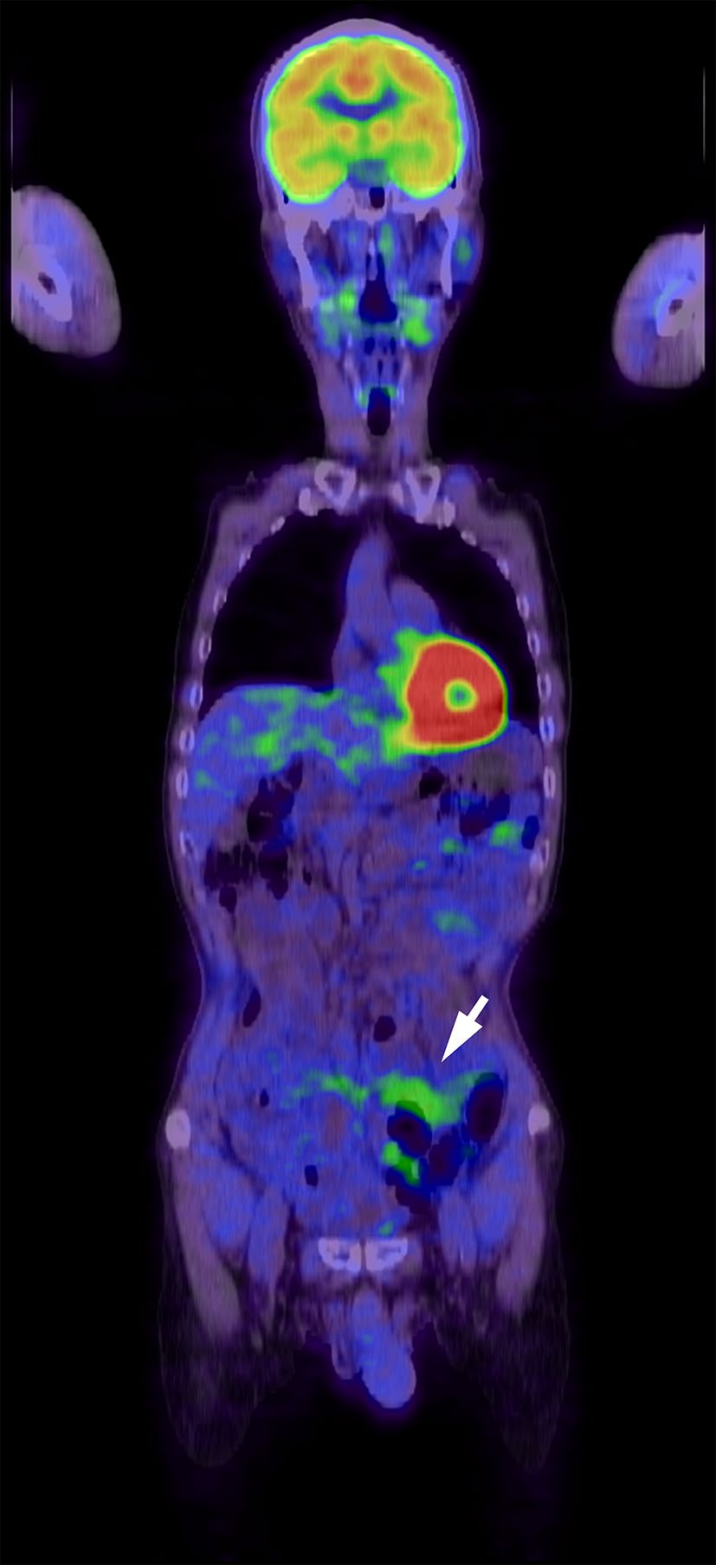
Whole-body FDG-PET imaging. Abnormal FDG uptake was not detected by PET in the patient’s body, including GI tract and lymph nodes. Arrow points to the enlarged small intestine

The patient’s vitals were within normal limits, and his physical examinations were benign. The initial laboratory data were only notable for albumin 3.4 g/dL and C-reactive protein 1.83 mg/dL, but the other routine blood cell count and biochemical investigations, including lactate dehydrogenase, were within normal reference ranges (Table 1). Repeat CT of abdomen and pelvis with or without contrast in our institution revealed diffuse abnormal thickening of the jejunum and the ileum with enlarged regional lymph nodes at the mesenteries (Fig. 1a, b). Repeat gastroscopy and colonoscopy performed in our institution were normal and were the same as the ones at the outside hospital. Subsequent pathological results of the random biopsies from the esophagus to the second portion of duodenum and from the terminal ileum to the rectum showed nothing significant including lymphoid infiltration. However, SBE revealed ceaseless villous atrophy throughout most of his jejunum and ileum without any mass, ulceration or stenosis (Fig. 2a–d). Histopathology of the SBE biopsy specimens from the jejunum and the ileum showed loss of villous architecture and significant infiltration of small cells in the lamina propria and the submucosal layers (Fig. 3a). Interestingly, the biopsy specimens from the distal third portion of duodenum did not show any clear endoscopic findings of villous atrophy using SBE but still consisted of the same lymphocyte infiltrate (data not shown). Microscopy under high power magnification revealed that these infiltrating mononuclear cells had relatively low nuclear grade, and the presence of lymphocytes in the epithelial layer were mostly normal (Fig. 3b). Immunohistochemistry (IHC, Fig. 4a–c) and flow cytometry (data not shown) showed that majority of such lymphocytes expressed CD3, CD5 and CD7 accompanied by aberrant CD20 expression, but not other B cell or natural killer (NK) cell-markers. In addition, IHC also showed that less than 10% of infiltrating cells expressed Ki-67 (Fig. 4d). These cells consisted of CD4^+^, CD8^−^, CD30^−^, CD56^−^, and Granzyme B^−^. In situ hybridization did not show Epstein–Barr virus-encoded RNA (data not shown). Additional analysis showed elevated soluble IL-2 receptor 1,470 unit/L, but anti-human T lymphotropic virus 1 antibody (Ab) was not detected (Table 1). Fluorodeoxyglucose-positron emission tomography (FDG-PET) detected diffuse small intestinal wall thickening and enlargement of mesenteric lymph nodes, but their maximal standardized uptake values were 1.4 and 2.8, respectively, which are considered as low as physiological uptake (Fig. 5). In addition, a bone marrow examination showed no infiltration with atypical lymphocytes. Peripheral T cell lymphoma, not otherwise specified (PTCL-NOS) of the GI tract was originally diagnosed based on the World Health Organization (WHO) Classification of Tumours of Haematopoietic and Lymphoid Tissues at the time [6], and the findings were considered to be stage II-2 based on the Lugano classification.

**Table 1:** Laboratory data

Hematology			Biochemistry			Serology	
WBC	7.4	× 10^3^/µL	TP	7.0	g/dL	HBs Ag	Negative
Neu	38.6	%	Alb	3.4	g/dL	HCV Ab	Negative
Lym	50.2	%	BUN	23	mg/dL	HTLV1 Ab	Negative
Mono	8.7	%	Cre	1.24	mg/dL		
Eosino	2.0	%	UA	5.9	mg/dL		
Baso	0.5	%	Na	142	mEq/L		
Aty Lym	0.0	%	K	4.7	mEq/L		
RBC	549	× 10^4^/µL	Cl	105	mEq/L		
Hb	16.4	g/dL	Ca	8.7	mg/dL		
Ht	48.4	%	LDH	167	U/L		
MCV	88.2	fl	AST	18	U/L		
MCH	29.9	pg	ALT	30	U/L		
MCHC	33.9	g/dL	γ-GTP	17	U/L		
Plt	19.5	× 10^4^/µL	ALP	225	U/L		
ESR	63	mm/hr	T Bil	0.6	mg/dL		
			TG	88	mg/dL		
			T Cho	140	mg/dL		
			Amy	109	U/L		
			Lip	35	U/L		
			Glu	119	mg/dL		
			HbA1c	5.6	%		
			CRP	1.83	mg/dL		
			CEA	2.8	ng/mL		
			sIL-2R	1470	U/mL

**Fig. 6:**
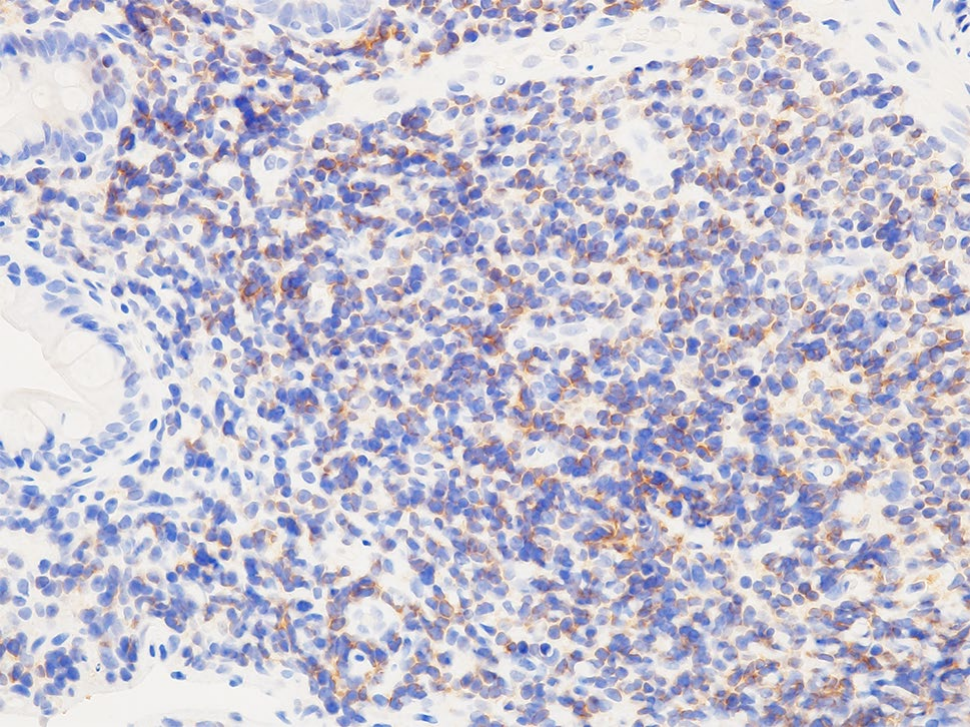
CCR4 expressions in the jejunum. The SBE specimens were applied for IHC with anti-CCR4 Ab

Chemotherapy was thus initiated and consisted of pirarubicin, cyclophosphamide, vincristine and prednisolone (THP-COP). However, there was no response in either clinical or histological presentations even after 6 cycles of THP-COP. Afterward, rituximab, an anti-CD20 Ab, was also administered to the patient. However, the symptoms persisted despite this therapy. Subsequently, the WHO classification was revised in 2016–2017, and associated with this, a revised diagnosis of indolent T cell lymphoproliferative disorder (T-LPD) of the GI tract was made for the patient [7–9]. Serial sections of SBE biopsy specimens revealed that the majority of infiltrating T cells in the tissue were surprisingly CCR4 positive (Fig. 6). Subsequently, mogamulizumab, an anti-CCR4 Ab, was administered to treat the patient, and all the patient’s symptoms including diarrhea and fever improved. To date, he is still followed at our institution, and he has remained in clinical remission for more than 2 years.

## Discussion

In this report, we presented a case of persistent diarrhea accompanied by wall thickening of small intestine where SBE was critical in the diagnosis of patient’s condition that otherwise would have persisted indefinitely.

Using SBE, we were able to appreciate villous atrophy in the jejunum and the ileum. Villous atrophy can be generally observed in the condition of chronic intestinal inflammatory disorders such as celiac disease, Crohn’s disease, eosinophilic gastroenteritis and familial Mediterranean fever. However, these are unlikely diagnosis since our patient is Asian, without any specific family history, and without histological findings of significant CD8^+^ T cell or eosinophil infiltrate. His prior history of anal fistula, however, would lead us to consider Crohn’s disease, but no other associated findings, such as ulceration, stenosis, abscess or granuloma were observed. Therefore, these inflammatory disorders were all ruled out.

With persistent diarrhea, intestinal wall thickening, and/or villous atrophy, neoplastic condition can also be considered. Enteropathy-associated T cell lymphoma (EATL), originally defined as type 1 EATL, and monomorphic epitheliotropic intestinal T cell lymphoma (MEITL), formerly designated type 2 EATL, are two distinct entities of primary intestinal T cell lymphomas with aggressive progression. Therefore, these are important to be included in the differential diagnosis. In our case, these two conditions were excluded, because histopathological assessment of our patient’s biopsy specimens showed normal-sized intraepithelial lymphocytes (IELs), and there were limited infiltration of small cells within lamina propria and submucosal layers. EATL and MEITL usually consist of intermediate to large-sized IELs with atypia and transmural infiltration with atypical lymphocytes. In addition, the immunophenotypical investigation of our patient revealed that the infiltrating cells were CD4^+^, CD5^+^, CD30^−^, CD56^−^ and Ki-67 index < 10%, which are different from the atypical lymphocytes in EATL and MEITL that are usually CD4^−^ and CD5^−^, cytotoxic, and high proliferation index with distinct markers such as CD56 and CD30. Moreover, the major symptom of our patient was mainly chronic diarrhea, and SBE showed no remarkable lesions other than villous atrophy in small intestine. On the other hand, clinical presentation of EATL and MEITL are generally acute because of high incidences of intestinal obstruction and perforation [10].

In our case, the histological and immunophenotypical diagnosis based upon the first SBE biopsies were done before WHO official definition of indolent T-LPD. Therefore, our diagnosis initially was PTCL-NOS, which is a heterogeneous category of mature T cell lymphomas without corresponding distinct entities. Afterward, the diagnosis of indolent T-LPD was re-made for our patient according to the revised WHO classification, that reclassified indolent lymphoproliferative disorders as issued in 2016 and 2017 [8, 9]. This corresponded to our patient’s chronic clinical presentation, lack of responsiveness for chemotherapy and relatively low histological aggressiveness with low Ki-67 index, as well as low nuclear grade of infiltrating lymphocytes.

The suggested first case of indolent T-LPD was initially described by Carbonnel et al. in 1994 [11]. Indolent LPD has been suggested to be associated with T cells, including CD4^+^, CD8^+^, CD4 CD8 double-positive (DP) and CD4 CD8 double-negative (DN) T cells, or NK cells [7, 10, 12–15]. So far, 28 cases of CD4^+^ T cell-, 17 cases of CD8^+^ T cell-, 1 case of DP T cell-, 1 case of DN T cell- and 24 cases of NK cell-associated indolent LPD have been reported. Those with detailed descriptions, there are 5 cases of CD4^+^ T cell-, 7 cases of CD8^+^ T cell-, and 3 cases of NK cell-associated indolent LPD with minor endoscopic lesions such as erythema/red mucosa, irregular granular/nodular mucosa and even normal mucosa without ulcer/erosion or polypoid/elevated lesions [11, 14–23]. In addition, the morbid lymphocyte infiltration in most of reported cases were spread throughout various parts of the GI tract mainly from the stomach to the colon. On the other hand, similar to our patient, some cases consisted of limited distribution of the morbid lymphocyte infiltration within the duodenum and the small intestine including 7 cases of CD4^+^, 4 cases of CD8^+^, and 1 case of DP T cell-associated indolent LPD [7, 12, 18, 19, 24, 25]. However, to the best of our knowledge, our patient is the first reported indolent T-LPD with endoscopic presentations observed by SBE, even though two previous papers, explaining the histological assessment of small intestines, have been published [16, 22].

To date, pathogenesis of indolent LPD is still not understood. Even though immune responses related to inflammatory diseases, such as celiac disease and Crohn’s disease, are speculated to also involve indolent T-LPD development, this has yet to be proven [7, 22, 25]. Although our patient had prior history of anal fistula, we do not think he has Crohn’s disease due to the lack of supportive endoscopic and histological findings from the entire GI tract. Since most of patients with indolent T-LPD primarily have the small intestine involvement, their symptoms of chronic diarrhea and weight loss usually lead to erroneous diagnosis such as celiac disease and Crohn’s disease [10].

Standard therapy for indolent T-LPD has not yet been established. Similar to our patient, variety of chemotherapy have been attempted in several prior reports without significant success [10]. Moreover, disease progression including extra-GI involvement and transformation to aggressive lymphoma had also been reported [16, 17, 22, 24, 25]. On the other hand, it has been reported that two patients receiving anti-CD52 Ab therapy demonstrated clinical remission [25]. In our patient, CCR4 expression in the infiltrating T cells was interestingly observed, and thus, treatment with mogamulizumab was attempted. Fortunately, this therapy resulted in clinical remission including improvement of diarrhea and increase of body weight. To date, we continue to observe for evidence of relapse. We have tried to review all the available publications regarding CCR4^+^ lymphoid neoplasms and adaptation of mogamulizumab treatment for such diseases as far as we could. However, we could not find any such published reports. In addition, we have also tried to find literatures where other antibody therapies were used for T cell neoplastic disorders. However, the report with anti-CD52 Ab described above was the only one we could find.

In conclusion, we presented a case of indolent T-LPD in the small intestine including the distal third portion of duodenum that was diagnosed by SBE with biopsies. Like our patient, many patients with chronic diarrhea are diagnosed as IBS when any infectious and inflammatory diseases are ruled out after extensive stool analysis and endoscopic exam. However, potentially serious diseases of the small intestine should be considered, such as lymphoid neoplastic conditions including T-LPD of GI tract. Therefore, the use of SBE with biopsies would be critical in accurate diagnosis of these conditions.

## Abbreviations

Ab: Antibody
DN: CD4 CD8 double negative
DP: CD4 CD8 double positive
EATL: Enteropathy-associated T cell lymphoma
FDG-PET: Fluorodeoxyglucose-positron emission tomography
GI: Gastrointestinal
H&E: Hematoxylin and eosin
IBS: Irritable bowel syndrome
IELs: Intraepithelial lymphocytes
IHC: Immunohistochemistry
MEITL: Monomorphic epitheliotropic intestinal T cell lymphoma
NK: Natural killer
PTCL-NOS: Peripheral T cell lymphoma-not otherwise specified
SBE: Single-balloon enteroscopy
THP-COP: Pirarubicin, cyclophosphamide, vincristine and prednisolone chemotherapy regimen
T-LPD: T cell lymphoproliferative disorder
WHO: The World Health Organization
